# 

**Published:** 1884-08-20

**Authors:** 


### EDITORIALS AND MISCELLANEOUS.

### EDITORIAL NOTICES.

NEW ADVERTISEMENTS.—See the new advertisement of Rio Chemical Company in this issue, referring to Celerina, Aletris Cordial, Acid Mannate and Pinus Canadensis.

SEE the advertisement of J. B. Daniel, Druggist, commencing with this number. This establishment is one of the strong and well-established Drug Houses of Atlanta. We commend it to the confidence and patronage of the Profession.

UNIVERSITY OF LOUISVILLE, MEDICAL DEPARTMENT.—We invite attention to the advertisement of the above medical institution, to be found in the present number of this Journal.

SOUTHERN MEDICAL COLLEGE.—Notice the advertisement of this Institution in this Journal.

SPECTACLES.—See the advertisement of Mr. Er Lawshe. Experienced in his business, reliable and clever, he is the man to trade with in the spectacle line.

THE PACIFIC MEDICAL JOURNAL and the Western Lancet have consolidated—a good idea.

THE CHICAGO MEDICAL JOURNAL is out in a new dress. We liked it before. We like it much better now.

CHICAGO MEDICAL SOCIETY.—We acknowledge the courtesy of an invitation to attend meeting of the above society, extended by Leston H. Montgomery, Secretary.

STEREOPTICON ILLUSTRATION.—This beautiful method of illustrating anatomy has been adopted in the Southern Medical College of Atlanta.

There is now, also, being fitted up an amphitheatre for the better convenienee and comfort of the students.

WORLD'S FAIR AT NEW ORLEANS.—A universal newspaper exchange has been opened in connection with the World's Fair. Hundreds of newspapers are received daily from all parts of the world and placed on file where all persons are cordially invited to inspect them. The press has performed a generous and kindly service to the Southern people by keeping the public mind impressed with the importance and magnitude of the great Exposition.

THE SOUTHERN MEDICAL RECORD has, by request, been forwarded to this great Exposition, and copies of it may be found on file for inspection by the brethren of the Profession, thousands of whom will, doubtless, visit this great display during the next winter.

*READ! READ!!*

☛ Many of our subscribers are in arrears. Friends, the aggregate of these small amounts is very important to us. Please let us hear from you at ONCE. R. C. WORD, Managing Editor.

### *RECEIPTED.*

1884.—Drs. M. D. Miles, S. W. Eaton, G. W. Earle to August, C. M. Bold, E. A. Speed, J. E. Wright, H. L. Norman, F. A. Small, J. W. McMichæl, A. B. Loving.
1883.—Drs. M. C. Mannaway, J. B. Morton, E. S. Strong, R. A. Fouche.
1885.—Drs. M. T. Bell, J. W. Miller, R. C. Fuller, T. L. Smoote, J. W. Harris.

